# An interview with Per Renstrom on his personal perspective of the changes and developments in the sports medicine field over the last 40 years

**DOI:** 10.1186/2052-1847-5-8

**Published:** 2013-04-29

**Authors:** Per Renstrom

**Affiliations:** 1 Flötviksvägen 51, Hässelby, 16572, Sweden

## How did you first become interested in sports medicine?

I grew up in a time before the existence of television, computers, and mobile phones. Children and adolescents had to occupy themselves with leisure time activities of their own choice and without the guidance of their parents. When I was very young I had the privilege of growing up in a small town in which participating in sport was the overall dominant activity for more or less everybody in my surroundings. I had some talent, so it was natural that sports activities swallowed me totally. I therefore developed a strong interest and working knowledge of some of the major sports.

After being accepted into the School of Medicine at University of Göteborg, Sweden it was natural for me to try to combine my very strong interest in sports with medicine. It was an early decision to look for courses in the area of Sports Medicine. The only problem was that it hardly existed as an independent entity at that time… in other words when I was ready to specialize, the discipline of Sports Medicine didn’t really exist! The closest and most developed area around 1970 was exercise physiology, so I started to work with some of the great scientists in this field such as Bengt Saltin and Gunnar Grimby. My first publications were with them in 1972.

With time, I realized I was better suited and more interested in working with sports orthopedics, which includes the use of surgery to treat sports injuries of the musculoskeletal system, and I was very fortunate to find a great individual and athlete with the same interest - Lars Peterson in Göteborg, Sweden. As a team, we strongly promoted Sports Medicine in the region and in the country. We were fortunate to be part of the rapid development of this area which occurred during the 1970´s and the 1980´s. We have always felt at home in the discipline of Sports Medicine as our expertise in sports had given us an edge in dealing with some of the issues faced. For example, we could understand the athlete´s terminology, the loads on the body endured through sport, the specific injury mechanisms, and so on.

## What do you think has been the main development in the field over the last 40 years?

Orthopedics have had an unbelievable development since 1970. In the last 40 years the main areas where there has been a “revolution” include the development of total joints, minimally invasive procedures such as arthroscopy, and the value of early motion, as well as the modern management of fractures. Sports Medicine has had a leading role especially in the development of arthroscopy and early motion.

## What is arthroscopy?

Arthroscopy as we know it was developed by Watanabe, from Japan [1], in the beginning of the 1960´s and it became a important diagnostic clinical tool in the 1970´s. It is a minimally invasive procedure alternatively known as “keyhole surgery” performed using the aid of a small operating telescope that is inserted into a joint through a small incision and can be used to examine and specifically treat damage to a joint. Arthroscopy in combination with video monitoring became one of the leading treatment tools in the 1980´s for the knee and later for the ankle, shoulder, elbow and wrist and recently also for the hip. Arthroscopy is now used for most joints and has resulted in safer surgery, decreased morbidity, and quicker rehabilitation, and most surgery since the 1990s is now carried out as outpatient surgery.

## Have there been other key developments that have improved treatment for athletes?

Concerning early motion, research by Tipton *et al.*[2] and Woo *et al.*[3] showed that motion was beneficial for all the tissues. Häggmark and Eriksson [4] showed that early motion and physiotherapy was possible and beneficial after anterior cruciate ligament (ACL) surgery of the knee and 5–10 years later the rest of the Sports Medicine community understood that this was the way to go. The concept of early motion has now been accepted by most medical disciplines.

During the beginning of the 1990´s van Mechelen *et al*., published the “Sequence of prevention” [5]. At the time, the Dutch colleagues involved in this initiated several conferences with a focus on prevention and I was involved in many of these discussions, helping me to understand the importance of prevention in sport. At this time the main challenge was to generate resources for research on prevention but that has changed dramatically for the better within the last 10–15 years. Other landmark contributions on prevention include Jan Ekstrand´s PhD thesis on injury prevention in football [6]and the foundation of F-MARC (FIFA) with their studies on football. During the last 10 years great work on prevention has been done in Norway, Canada, Australia and many other places.

## How has technological innovation contributed to the field of sports medicine over the last 40 years and how can we further benefit from it in the future?

During the 1970s there was a rapid development of the use of arthroscopy of the knee allowing us to make correct diagnoses, but at the time we then had to look through the arthroscope without having a screen. During the 1980s there was an explosion in the diagnosis of new injuries and in the 1990s and thereafter, in developing new surgical techniques and equipment.

When the video screen was developed at the end of the 1970´s we could start to treat injuries inside the knee, with arthroscopic ACL reconstruction starting to take off in the 1980s, becoming common during the 1990s. Arthroscopic ACL reconstruction was used by many surgeons during the 1990´s and out-patient ACL surgery was also introduced. During the last 10 years the development has been mostly focused on the technique of double bundle ACL surgery and improving the ACL surgery techniques refining the details. It has become evident that it takes experience to become a successful ACL- surgeon and that this surgery should probably be more centralized.

The development of osteoarthritis after an ACL injury depends very much on the injury mechanism and concurrent meniscus injury. During the early time of arthroscopy the importance of saving the meniscus became increasingly understood. Different types of meniscus suture techniques have been developed over time and used in conjunction with improved technical developments.

Surgery of the shoulder joint exploded during the 1980´s with many new diagnoses and in the 1990´s many new instruments and new diagnostic tests were designed. Most surgery undertaken in present times is arthroscopic and continues to be a challenge.

Hip arthroscopic surgery has rapidly developed during the last 10 years. The management of FAI- femuro-acetabular impingement is a major step forward. As with any new idea, many people jump on the bandwagon but it is becoming increasingly evident that this is difficult surgery and should be centralized to specialists. The challenge over the next few years will be to refine the indications for hip arthroscopy and the role of subsequent hip arthroscopic surgery.

## Why do you think the field is growing so much and which direction do you see it taking in the future?

Recently, there has been an explosion of general interest in sports. There is an increased interest in improving health resulting from an increased participation in sports activities - especially running and other long distance activities. Also, with the increasing amount of sports channels offered in the last 5–10 years, people extensively watch sports on television. In parallel with this, there is more mass-media coverage of different sports available, and sports have, for better or for worse, become an area with large financial interests at stake. This growth in popularity has had the knock on effect that people are inevitably interested in how to maximise the chances of athletes competing at their best i.e. by both preventing injuries and effectively treating those injuries that occur.

People appreciate that an injured athlete (who is often a vital investment) cannot participate if he/she is injured. Football clubs for example are now providing sports medicine management and ensuring that there are more resources available to prevent injury in the first place and this is a welcome development.

It can also be said that Sports Medicine is stimulating and often fun, making this discipline attractive. It is very rewarding to be a member of a sport´s medical team and the sports physician often deals with motivated and otherwise healthy patients. The interaction is mostly quite positive and the end result is often a very satisfied patient.

It is interesting to note that the few of us that were active in Sports Medicine in the 1970´s and 1980’s mostly had to fight to get resources and to be seen. We had to work against lots of scepticism in our departments. We learned to fight for what we believed in and to get resources for our field at that time, but it seems that good work and persistence has prevailed in the end.

## What challenges and developments can we expect to see in the next few years in sports medicine, and how can research contribute to meeting these challenges?

Many people want to be part of Sports Medicine so it will be a challenge to keep up the quality and use of evidence-based medicine. Some may try to take shortcuts to fame. I believe that dedication to hard work, good education and involvement in research will be the main tenets that determine how well Sports Medicine will develop as a discipline.

There is still a lack of resources going to the sports medical service and research today. Available financial resources in the dominant sports are mainly allocated to attract good athletes. It is a fact that an athlete with an injury cannot fully compete and is then not very valuable. The smart coaches of today understand this and support Sports Medicine service of highest quality.

Effective treatment of an injury must be evidence-based, or if the evidence is not there at least be based on long-term experience. The largest problem in most team sports is an ACL injury, with a substantial risk for re-injury, complications, and/or osteoarthritis, especially if the athlete’s return to sport is too early. Top level sport in itself is a risk factor. Today there are indications for sustained healing of knee articular cartilage for as long as 1–2 years after an ACL injury. This is a new challenge for both the surgeon and the rehabilitation team, and will affect the rehabilitation and time to return to sport. The good news is that ACL programs can be effective. Pooled estimates suggest a substantial beneficial effect of ACL injury prevention programs, with a risk reduction of 52 % in female athletes and 85 % in male athletes. The individual coaching of the player by the team physiotherapist and compliance with the training program by the player are key factors in the rehabilitation.

Tendinopathy in Achilles, patellar and rotator cuff tendons remain to be major challenges in sports as they are difficult to manage and take a long time to return to sport. New techniques addressing tendon management such as tissue engineering and tissue regeneration seem promising. These methods include molecular approaches by which genetically modified cells, including stem cells, synthesize growth factors or other mediators needed for progression of failed healing. However, molecular procedures are not yet ready for routine clinical use. Novel mini-invasive procedures that target underlying pathology, such as abnormal neoinnervation, are being developed and while initially promising, still necessitate high quality randomized controlled trials before management of tendinopathy can be recommended.

## What will the new *BMC Sports Science, Medicine, and Rehabilitation* bring to the sports medicine community?

Sports Medicine by it’s very nature is multidisciplinary and is defined differently around the world. It is mostly dominated by orthopaedic surgery including rehabilitation, return to sports, and prevention, but often it also includes exercise medicine, internal and general medicine, pediatrics, infection, and gynecology. The new *BMC Sports Science, Medicine, and Rehabilitation* with its broad scope and inclusive editorial policy will offer a home for diverse research in this area.

## What impact does ‘openness’ - in the form of open access journals and open peer review - have within the sports medicine field?

It should be increasingly popular providing you publish good quality research and have rigorous peer review. I believe that open access journals and open peer review will be part of the future although it may take time for people to change their habitual behaviours. The field of sports science and medicine is unquestionably an area with a very high public interest. The transparent open peer review process on *BMC Sports Science, Medicine & Rehabilitation* provides greater trust in the research you report. The fact that the research published is open access also increases the impact of the scientific findings by making them widlely available to the general public.

## Author’s information

Per Renstrom, PhD, is an Emeritus Professor at the Karolinska Institutet in Sweden and is a well-respected figure in the field of orthopedic sports medicine. His particular interests are in surgery, rehabilitation, prevention of sports injuries, biomechanics of knee and ankle ligaments, and in particular the healing process of injuries to the Achilles tendon. He has co-authored over 170 original scientific publications and in this article we find out a little more about his thoughts on how the sports medicine field has changed during his time as an active researcher and member of various sports societies such as president of ISAKOS -the International Society of Arthroscopy, Knee Surgery and Orthopedic Sports Medicine, vice president of FIMS - International Sports Medicine Federation, member of the Medical Commission of the IOC -International Olympic Committee and member of the Sports Science and Medical Commission of the ITF -International Tennis Federation as well as of the ATP World Tour.

## Pre-publication history

The pre-publication history for this paper can be accessed here:

http://www.biomedcentral.com/2052-1847/5/8/prepub
